# Physical Characteristics of a Citrullinated Pro-Filaggrin Epitope Recognized by Anti-Citrullinated Protein Antibodies in Rheumatoid Arthritis Sera

**DOI:** 10.1371/journal.pone.0168542

**Published:** 2016-12-21

**Authors:** Nicole Hartwig Trier, Bettina Eide Holm, Ole Slot, Henning Locht, Hanne Lindegaard, Anders Svendsen, Gunnar Houen

**Affiliations:** 1 Department of Autoimmunology and Biomarkers, Statens Serum Institut, Artillerivej, Copenhagen S, Denmark; 2 Department of Rheumatology, Glostrup Hospital, Nordre Ringvej, Glostrup, Denmark; 3 Department of Rheumatology, Frederiksberg Hospital, Nordre Fasanvej, Frederiksberg, Denmark; 4 Department of Rheumatology, Odense University Hospital, Søndre Boulevard, Odense C, Denmark; 5 Department of Epidemiology, Biostatistics and Bio-demography, Institute of Public Health, University of Southern Denmark, Campusvej, Odense M, Denmark; Duke University School of Medicine, UNITED STATES

## Abstract

Rheumatoid arthritis (RA) is an autoimmune disease of complex etiology. A characteristic feature of a subset of RA is the presence of anti-citrullinated protein antibodies (ACPA), which correlate with a progressive disease course. In this study, we employed streptavidin capture enzyme-linked immunosorbent assay to analyze ACPA reactivity. Using the pro-filaggrin peptide HQCHQEST-Cit-GRSRGRCGRSGS, as template, we analyzed the reactivity of RA sera and healthy donor sera to various peptides in order to determine the physical characteristics of the citrullinated pro-filaggrin epitope and to examine whether biotin labelling influence antibody recognition. The full-length cyclic pro-filaggrin peptide and a linear form with a N-terminal biotin, was recognized to the same level, whereas, a notable difference in ACPA reactivity to the linear peptides with a C-terminal biotin was found, probably due to steric hindrance. Screening of linear and cyclic truncated peptides, revealed that small cyclic peptides containing 10–12 amino acids are favored over the linear. Moreover, the charged amino acids C-terminal to citrulline were found to be essential for antibody reactivity, most important was the charged amino acid in position 4 C-terminal to citrulline. Collectively, peptide structure, length, the presence of charged amino acids and biotin labelling markedly influence antibody reactivity. In relation to the clinical diagnostics of ACPA, these findings may reflect the differences in diagnostic assays used for detection of ACPA, which relates to differences in sensitivity and specificity dependent on the assay applied.

## Introduction

Rheumatoid arthritis (RA) is an autoimmune disorder characterized by synovial joint inflammation. RA affects 1–2% of the world’s population with a female preponderance of 3:1 [1–2]. Onset of the disease is most frequent between the ages of 40 to 50 [3].

RA is diagnosed according to clinical manifestations supported by detection of the autoantibodies; the rheumatoid factor (RF) and anti-citrullinated protein antibodies (ACPA) [4]. RFs, directed to the Fc regions of IgG molecules, are detected in 50–80% of RA sera, but are also associated with other diseases and can be found in healthy individuals (10–30%), lowering their specificity for RA and limiting their diagnostic usefulness [5–8]. ACPAs, being detected in 60–80% of RA sera, are more specific for RA, as they are rare in other diseases and only present in approximately 2% of the healthy population [9–11]. ACPAs as well as RFs, have been detected in the serum of RA patients years before the onset of RA [12–14], suggesting that the development of RA occurs long before the appearance of symptoms [15–17]. In addition, high ACPA titers have been observed in RA patients approaching disease onset and only very few RA patients starts generating ACPA after onset of their symptoms [18]. The occurrence of ACPA-positive RA is linked with genetic risk factors, such as the protein tyrosine phosphatase N22 and the MHC class II alleles that predispose for RA [18–21]. In addition, smoking and bacterial infections have been suggested to cause citrullination of autoantigens and hence induce the generation of ACPA [22–23].

Even though specific for RA, the presence ACPA does not reveal the underlying antigen specificities that initiate and/or perpetuate inflammatory autoimmune reactions. In fact, several citrullinated autoantigens have been identified, such as vimentin, α-enolase, fibrinogen and collagen II [24–28]. The process behind the generation of citrullinated autoantigens in the joints is not evident, however, the actual citrullination is catalyzed by a family of calcium-dependent enzymes, the peptidyl arginine deiminases [29].

Detection of ACPAs was originally described by Schellekens *et al*. Girbal-Neuhauser *et al*, Simon *et al* and Sebbag *et al* [10,30–32]. Currently, ACPA are most commonly detected by reactivity against cyclic citrullinated peptides (CCP) in an enzyme-linked immunosorbent assay with immobilized CCPs [33]. The precursor for this assay, CCP1, was originally described by Schellekens *et al* [9]. Using a synthetic citrullinated 19mer pro-filaggrin peptide (CCP1, HQCHQEST-Cit-GRSRGRCGRSGS), comprising amino acid residues 306–324 of filaggrin, Schellekens *et al* demonstrated that specificity and sensitivity were increased when employing cyclic peptides [9]. Apparently, the cyclic form allowed the citrullinated epitope to be optimally exposed. Cyclic peptides were used to mimic the original structure found within the pro-filaggrin protein due to structural features, as short peptides usually do not have a preferential conformation in solution [34–35]. Analysis of antibody-peptide complexes has shown that peptides often adopt a β-turn structure within the complex [36–37], a motif which frequently is encountered within the filaggrin sequence [29]. Thus, peptides were cyclized in order to force the peptide into a β-hairpin conformation, as cysteine-bridged cyclic peptides previously have been shown to mimic the β-turn structure of an epitope and bind with enhanced affinity to antibodies [38].

Based on the successful application of cyclic peptides by Schellekens *et al*, peptide cyclization has been employed in an attempt to improve antigen presentation [9,39–40]. However, the positive effect of cyclization was not found for all peptides, indicating that other factors influence antibody reactivity. Studies by Kobylyansky *et al*, analyzing antibody reactivity to a filaggrin peptide (amino acids 228–245), demonstrated that peptide cyclization had no influence on reactivity and sensitivity [39]. Identifying a reactive region in amino acid residues 228–245, a cyclic and a linear peptide version were generated, however the two peptides showed similar characteristics [39]. These findings are in accordance to studies by Trier *et al*, describing the cross-reactivity of a human IgG_1_ anti-citrullinated fibrinogen monoclonal antibody to cyclic and linear CCP1-derived truncated peptides [41].

In this study, we examined the physical characteristics of a citrullinated pro-filaggrin epitope using RA sera in a streptavidin capture enzyme-linked immunosorbent assay (ELISA) using the original pro-filaggrin sequence CCP1 (HQCHQEST-Cit-GRSRGRCGRSGS) with special emphasis on peptide structure, peptide length and peptide presentation.

## Materials and Methods

### Peptides and peptide synthesis

Peptides applied were obtained from Schäfer-N (Lyngby, Denmark) and synthesized using standard Fmoc solid-phase peptide synthesis as previously described [42]. Cyclization of peptides was performed by traditional air oxidation. Following oxidation, all peptides were purified by reverse-phase high-performance liquid chromatography and peptide identity was confirmed with liquid chromatography-mass spectrometry as previously described and illustrated [41]. The peptides applied are listed in Table 1.

**Table 1:** Pro-filaggrin peptides applied for reactivity screening

Peptide	Amino acid sequence
*LCP/CCPa	HQCHQEST-Cit-GRSRGRCGRSGS
*LCP/CCPb	CHQEST-Cit-GRSRGRC
*LCP/CCPc	CQEST-Cit-GRSRGC
*LCP/CCPd	CEST-Cit-GRSRC
*LCP/CCPe	CST-Cit-GRSC
*LCP/CCPf	CST-Cit-GRC
*LCP/CCPg	HQCHQEST-R-GRSRGRCGRSGS
*[dA^**8**^**]LCPb**	CHQEST-Cit-**dA**RSRGRC
**[G**^**13**^**,G**^**15**^**,G**^**18**^**]CCPa**	HQCHQEST-Cit-GRS**G**G**G**CG**G**SGS
**[G**^**11**^**,G**^**13**^**,G**^**18**^**]CCPa**	HQCHQEST-Cit-G**G**S**G**GRCG**G**SGS
**[G**^**11**^**,G**^**13**^**,G**^**15**^**]CCPa**	HQCHQEST-Cit-G**G**S**G**G**G**CGRSGS
**[G**^**11**^**,G**^**13**^**]CCPa**	HQCHQEST-Cit-GRSRG**G**CG**G**SGS
**[K**^**13**^**]CCPa**	HQCHQEST-Cit-GRS**K**GRCGRSGS
**[K**^**11**^**,K**^**13**^**,K**^**15**^**]CCPa**	HQCHQEST-Cit-G**K**S**K**G**R**CGRSGS

* tested with an N- and a C-terminal biotin labelling

### Patient samples

20 RA sera diagnosed according to the American College of Rheumatology (ACR) classification criteria were analyzed in this study [43]. The sera were evaluated with respect to RF levels and anti-CCP2 concentrations. Sera from individuals with RA were obtained from the Department of Rheumatology, Glostrup Hospital, Department of Rheumatology, Frederiksberg Hospital, Department of Rheumatology, Odense University Hospital and Epidemiology, Biostatistics and Bio-demography, Institute of Public Health, University of Southern Denmark. Healthy controls were obtained from the biobank at Statens Serum Institut http://www.nationalbiobank.dk/.

### Ethics statement

The authors were not involved in drawing blood or collecting of samples, which were collected as part of routine medical care, however, the authors treated the patients of this study. In this retrospective study, the samples were blinded prior to laboratory analyses, thus the sera were used anonymously and no written consent was necessary. The study was approved by all the scientific ethics committees in Denmark (Project ID:19980024 PMC and H-15009640).

## Detection of antibodies by streptavidin capture enzyme-linked immunosorbent assay

96-well maxisorp microtiter plates (Nunc, Roskilde, Denmark) were precoated with streptavidin (Sigma Aldrich, St Louis, Mo, USA) diluted in carbonate buffer (15 mM Na_2_CO_3_, 35 mM NaHCO_3_, 0.001% phenolred, pH 9.6) (SSI Diagnostica, Hillerød, Denmark) to 1 μg/mL for 2 hours at room temperature (RT) followed by coating with overlapping biotinylated EBNA-1 peptides diluted in PBS (10 mM Na_2_HPO_4_/NaH_2_PO_4_, 0.15 M NaCl, pH 7.2) (Statens Serum Institut) to 1μg/mL for 2 hours at RT. Sera diluted 1:200 in Tris-Tween-NaCl (TTN) buffer (0.05 M Tris, 0.3 M NaCl, 1% Tween 20, pH 7.4) (SSI Diagnostica, Hillerød, Denmark) were then incubated in duplicates for 1 hour at RT. After washing with TTN buffer, alkaline phosphatase (AP)-conjugated goat-anti human IgG, IgA or IgM (Sigma Aldrich, St Louis, Mo, USA), diluted in TTN to 1 μg/mL, was added to the wells and the plates incubated for 1 hour at RT. For quantification of bound Abs, AP activity was determined with *p-*nitrophenylphosphate (Sigma Aldrich, St Louis, Mo, USA) (1 mg/mL) diluted in AP substrate buffer (1M diethanolamine, 0.5 mM MgCl_2_, pH 9.8) (SSI diagnostica, Hillerød, Denmark). The absorbance was measured at 405 nm, with background subtraction at 650 nm, using a Thermomax microtitre plate reader (Molecular Devices, Menlo Park, CA, USA). Samples were corrected for non-specific reactivity in non-coated wells.

### Competitive inhibition assay

The pro-filaggrin peptide CHQEST-Cit-GRSRGRC (1 μg/mL) in a linear and a cyclic form was coated onto the surface of the wells of Maxisorp microtitre plates diluted in carbonate buffer overnight at 4°C. All incubations with antibodies diluted in TTN were carried out for 1 h at RT followed by 3 washes in TTN buffer. Following incubation, peptide analogues (1 mg/mL) and RA sera (200 fold dilution) were added to the microtiter plate and incubated. AP-conjugated goat anti-human IgG (1 μg/mL) was used as secondary antibody. Antibody level was quantified using *p*NPP (1 mg/mL) diluted in AP substrate buffer. The absorbance was measured as previously described.

### Statistical analysis

Statistical calculations were performed using two measurements of 20 RA sera and 20 healthy control sera. The values obtained in this study were compared further by using the two-tailed Student’s t-test for single column analysis and ANOVA applying Dunnets test, which compared all columns to control columns.

## Results

### Reactivity of Rheumatoid Arthritis sera to citrullinated pro-filaggrin peptides

In order to compare ACPA reactivity to the pro-filaggrin peptide originally employed for determination of ACPA in a streptavidin capture ELISA, we screened 20 RA sera and 20 sera from healthy donors for reactivity to biotinylated cyclic and linear versions (**LCPa**, **CCPa**) and non-citrullinated controls (**LCPg**, **CCPg**).

**Fig 1:**
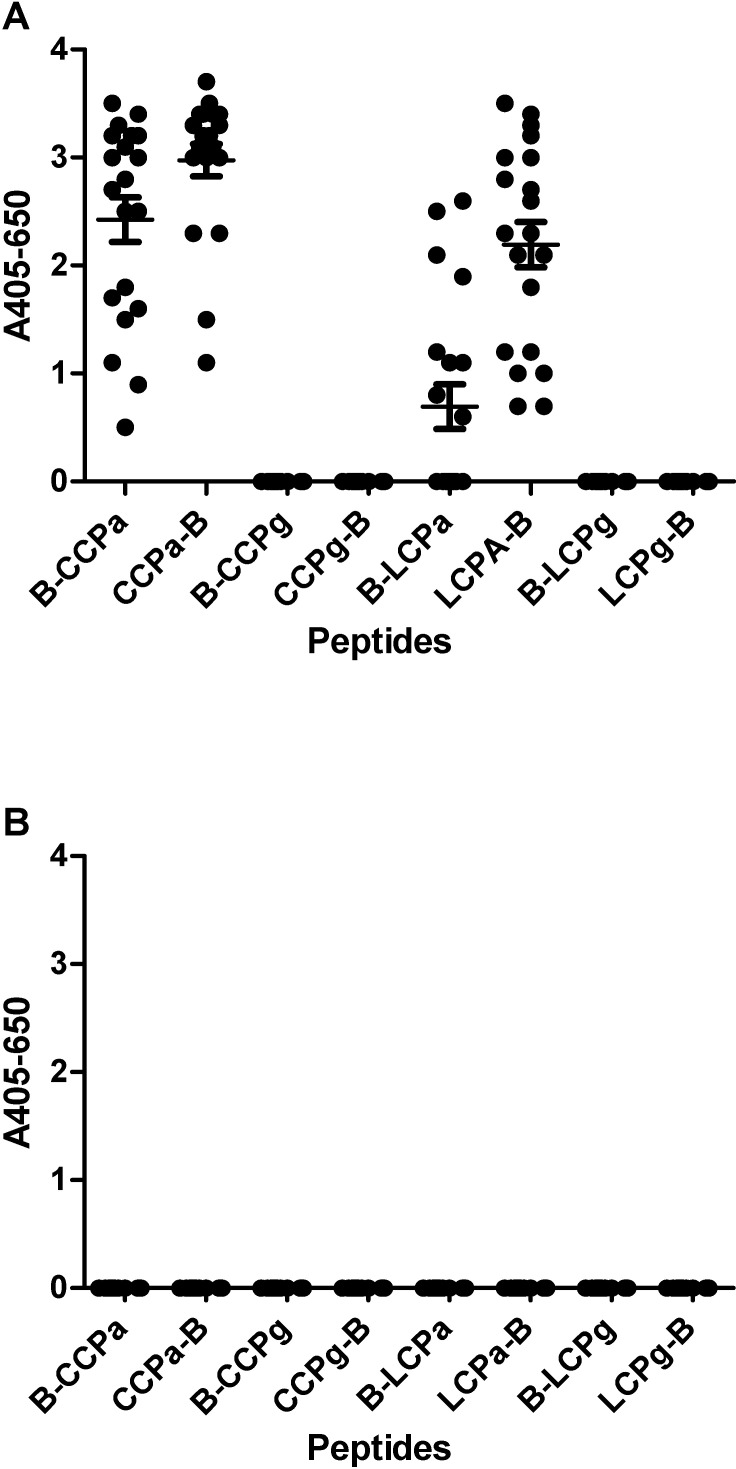
Reactivity of RA sera (n = 20) and healthy donor sera (n = 20) to linear and cyclic citrullinated peptides pro-filaggrin peptides analyzed by streptavidin capture ELISA. The peptide HQCHQEST-Cit-GRSRGRCGRSGS was used as template, whereas the non-citrullinated peptide was used as control. B-LCPa and LCPa-B represent peptides with a N- and C-terminal biotin labelling, respectively. A. Reactivity of RA sera to pro-filaggrin peptides. B. Reactivity of healthy donor sera to pro-filaggrin peptides.

Fig 1 illustrates the reactivity of RA sera and healthy donor sera to the pro-filaggrin peptides. As seen, the RA sera showed significant reactivity to **LCPa** and **CCPa** (*p* = <0.0001) compared to the non-citrullinated control peptides (**LCPg**, **CCPg**). No specific reactivity was found when screening healthy donor sera for reactivity (Fig 1B). As seen (Fig 1A), a notable difference in antibody reactivity was found between the peptides containing an N-terminal and a C-terminal biotinylation. In general, the linear peptides containing a C-terminal biotinylation experienced an increased antibody reactivity, compared to the N-terminal biotinylated version.

### Reactivity of RA sera to cyclic and linear citrullinated pro-filaggrin peptides

Previous studies describing reactivity of a human monoclonal antibody to citrullinated pro-filaggrin peptides indicated that antibody reactivity to these peptides is dependent on peptide conformation and peptide length [41]. To determine whether these results relate to RA patient sera as well, the reactivity of RA sera to cyclic and linear truncated peptides were analysed by streptavidin capture ELISA. Moreover, linear peptides containing both N- and C-terminal biotinylations were screened for reactivity, as early findings (Fig 1) indicated a notable difference in antibody reactivity dependent on the location of biotin labelling.

**Fig 2:**
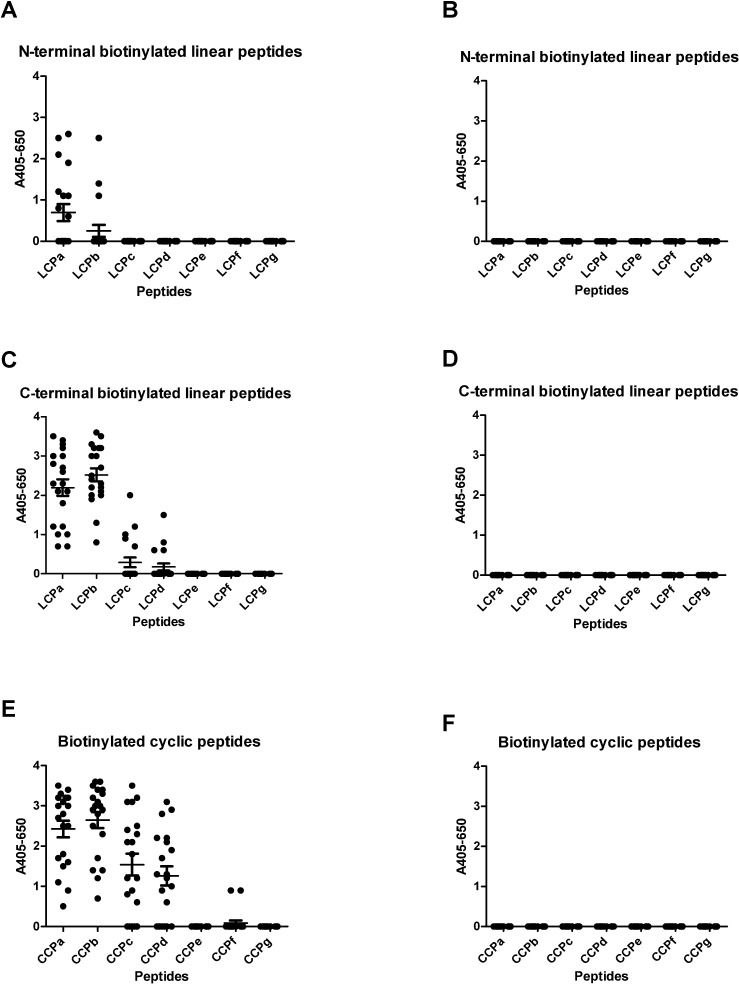
Reactivity of RA sera (n = 20) and healthy donor sera (n = 20) to linear and cyclic citrullinated pro-filaggrin peptides analyzed by streptavidin capture ELISA. The peptide HQCHQEST-Cit-GRSRGRCGRSGS was used as template, whereas the non-citrullinated peptide was used as control. A. Reactivity of RA sera to N-terminal biotinylated linear peptides. B. Reactivity of healthy donor sera to N-terminal biotinylated linear pro-filaggrin peptides. C. Reactivity of RA sera to C-terminal biotinylated linear peptides. D. Reactivity of healthy donor sera to C-terminal biotinylated linear pro-filaggrin peptides. E. Reactivity of RA sera to cyclic peptides. F. Reactivity of healthy donor sera to cyclic pro-filaggrin peptides.

Fig 2 illustrates the reactivity of RA sera to linear and cyclic truncated pro-filaggrin peptides. As seen in Fig 2A and 2C, the RA sera reacted with the linear peptides **LCPa**-**LCPb** containing a N-terminal biotin and the linear peptides **LCPa**-**LCPd** containing a C-terminal biotin, although only significant reactivity to **B-LCPa** and **LCPa-B** and **LCPb-B** was found (*p* = <0.0001). In contrast, significant antibody reactivity was found to the cyclic peptides **CCPa**-**CCPd** (*p* = <0.0001). No specific reactivity was found when screening healthy donor sera for reactivity (Fig 2B, 2D and 2F**)**. These findings indicate the peptide presentation is essential for antibody reactivity. Again, peptides with C-terminal biotin were generally better than peptides with N-terminal biotin.

### Reactivity of RA sera in competitive inhibition assay

Next, the inhibitory effect of the linear and cyclic peptides **CCPa**, **CCPb**, **LCPa** and **LCPb** was determined by competitive inhibition assays.

**Fig 3:**
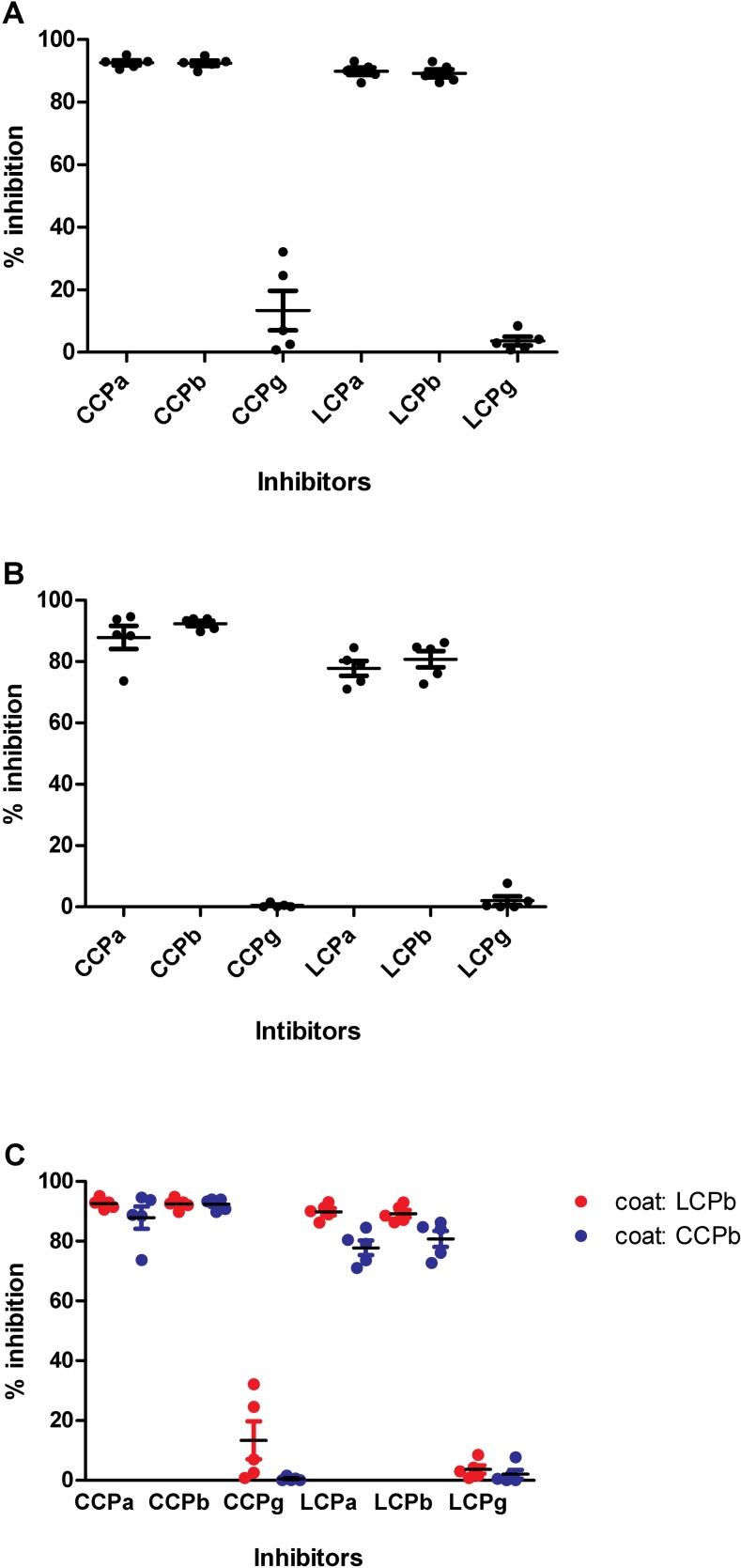
Reactivity of RA sera (n = 5) to cyclic and linear pro-filaggrin analyzed in a competitive inhibition assay. The linear and cyclic citrullinated peptides CHQEST-Cit-GRSRGRC were coated onto the wells. CCPa, CCPb, CCPg, LCPa, LCPb and LCPg were used as inhibitors. A. Reactivity of RA sera to LCPb. B. Reactivity of RA sera to LCPb. C. Comparison of ACPA reactivity to LCPb and CCP.

Fig 3 illustrates the inhibitory effect of the cyclic and linear pro-filaggrin version on the reactivity of RA sera to **LCPb** and **CCPb**. As seen, the linear and the cyclic peptides were very efficient in inhibiting antibody reactivity to **LCPb** and **CCPb** (**Fig 3A and 3B**), whereas the non-citrullinated controls (**CCPg** and **LCPg**) were not able to inhibit antibody reactivity, although a few RA sera occasionally were inhibited for ACPA reactivity to the **LCPb** peptide by the control peptide **CCPg** (Fig 3A). As seen, the full-length peptides **LCPa**/**CCPa** and the truncated peptides **LCPb**/**CCPb** inhibited antibody reactivity to the same extent. Comparison of the inhibitory effect of the cyclic and linear peptides (Fig 3C) showed a significant difference in inhibition of the LCPa peptide ((*p* = <0.0238) to the **LCPb** peptide relative to the cyclic version. Besides from this, these findings indicate that the linear and cyclic versions inhibit antibody reactivity to the same level, confirming that peptides in solution/free form behave similar to the screenings earlier described in Fig 2. In addition, these findings indicate that the poor reactivity to the citrullinated linear peptides containing a N-terminal biotin is directly ascribed to the location of the biotin labelling and the possible effect of steric hindrance.

### Reactivity of RA sera to cyclic and linear citrullinated pro-filaggrin peptides

Screening of citrullinated truncated linear and cyclic peptides revealed that peptide conformation is essential for antibody reactivity. In order to analyse this further, we analysed the reactivity of RA sera to a substituted peptide, where the Gly residue next to Cit was replaced with a D-amino acid. Generally, the incorporation of D-amino acids into the polypeptide chain imposes local conformational constraints [44].

**Fig 4:**
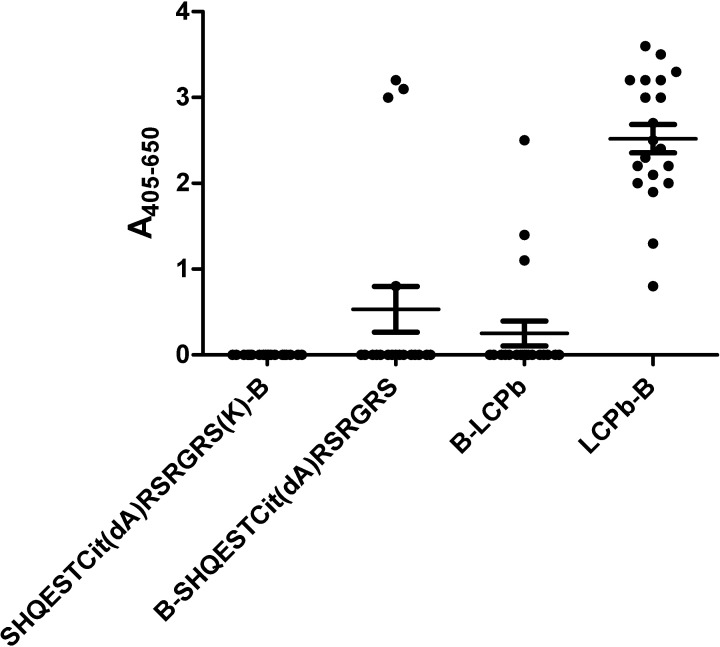
Reactivity of RA sera (n = 20) to D-Ala substituted peptides analyzed by streptavidin capture ELISA. The linear peptide SHQEST-Cit-GRSRGRS was used as template, LCPb was used as control. D-Ala substitution was introduced at position 8.

Fig 4 illustrates the reactivity of RA sera to **LCPb** and the substituted peptides **[dA**^**8**^**]LCPb** with N/C-terminal biotinylations. As seen, notable reactivity was found to the control **LCPb-B**, whereas no reactivity was found to the substituted version **[dA**^**8**^**]LCPb**. In contract, no notable difference in antibody reactivity was found to the substituted **B-LCPb** version compared to the control **B-LCPb**, probably because limited reactivity is found to the control. These findings confirm that the peptide conformation is essential for antibody reactivity.

### Reactivity of RA sera to substituted pro-filaggrin peptides

Thorough analysis of citrullinated epitopes reveal that peptide backbone primarily in combination with a Cit-Gly motif is essential for reactivity, although occasionally other amino acids are tolerated C-terminal to Cit [41]. Screenings of several epitopes prior to this study indicated that especially charged amino acids C-terminal to Cit are essential for antibody reactivity (data not shown) [41]. In order to examine this further, we analyzed the reactivity of RA sera to Arg -depleted pro-filaggrin peptides by streptavidin capture ELISA.

**Fig 5:**
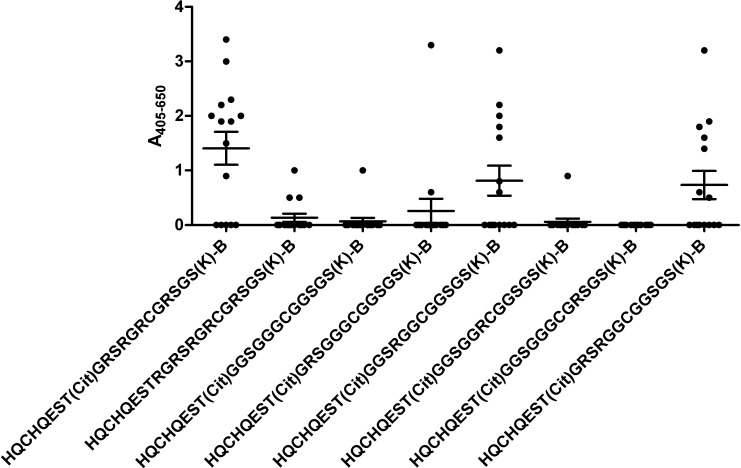
Reactivity of RA sera (n = 20) to glycine-substituted linear citrullinated pro-filaggrin peptides analyzed by streptavidin capture ELISA. The citrullinated peptide HQCHQEST-Cit-GRSRGRCGRSGS was used as template. Non-citrullinated LCPg was used as negative controls.

Fig 5 illustrates the reactivity of RA sera to Arg-depleted pro-filaggrin peptides. As seen, the peptide completely depleted of Arg **[G**^**11**^**,G**^**13**^**,G**^**15**^**,G**^**18**^**]CCPa** was not recognized significantly by the RA sera relative to the control peptide. Neither were the peptides containing a single Arg in positions 11, 15 and 18. In contrast, the peptides **[G**^**11**^**,G**^**15**^**,G**^**18**^**]CCPa** and **[G**^**11**^**,G**^**13**^**]CCPa** containing a single Arg in position 13 and in position 11 and 13, respectively, were significantly recognized by the RA sera (*p* = <0.0321). However, as the average ACPA reactivity to the peptides **[G**^**11**^**,G**^**15**^**,G**^**18**^**]CCPa** and **[G**^**11**^**,G**^**13**^**]CCPa** was comparable, the ACPA reactivity observed to the two peptide is primarily ascribed to the presence of Arg in position 13, corresponding to Arg in position 4 C-terminal to Cit.

To determine whether the effect of a charged amino acid is restricted to the presence of Arg in the citrullinated epitope, we replaced Arg with Lys at relevant positions.

**Fig 6:**
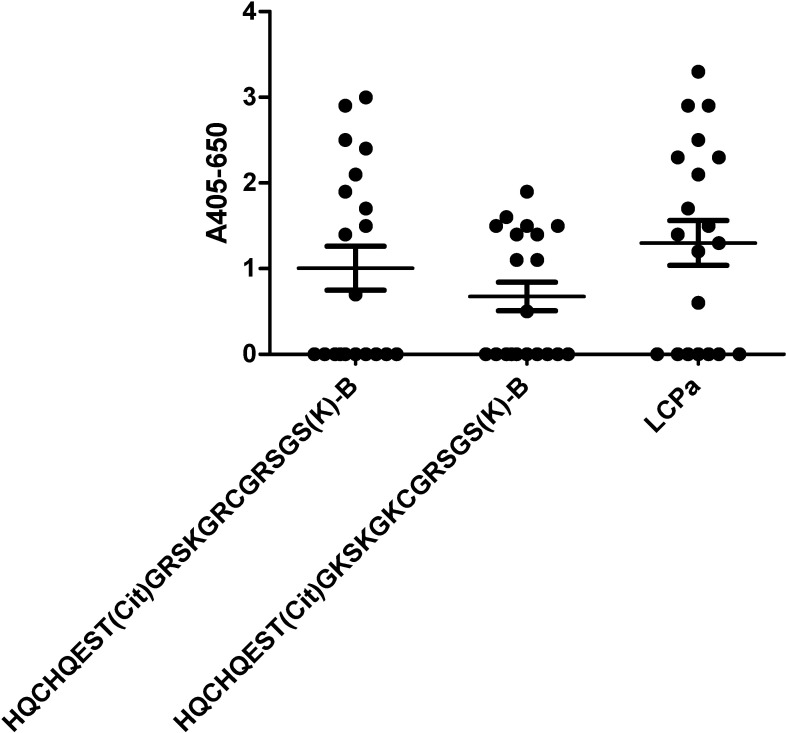
Reactivity of RA sera (n = 20) to lysine-substituted linear citrullinated pro-filaggrin peptides analyzed by streptavidin capture ELISA. The citrullinated peptide HQCHQEST-Cit-GRSRGRCGRSGS was used as template. LCPa was used as positive control.

Fig 6 illustrates the reactivity of RA sera to substituted pro-filaggrin peptides analyzed by streptavidin capture ELISA. As seen, a small reduction, however not significant, in ACPA reactivity was found to the pro-filaggrin peptide **[K**^**13**^**]CCPa** relative to the control peptide **CCPa**, where the essential Arg in position 13 was replaced with Lys. When substituting the remaining Arg residues in position 11 and 15, **[K**^**11**^**,K**^**13**^**,K**^**15**^**]CCPa**, a notable reduction in antibody reactivity was found. These findings indicate that although the presence of Arg in position 13 is essential, its presence is not critical, other charged amino acids are adequate as well.

To confirm this further, we searched the literature, describing reactivity of RA sera to citrullinated epitopes, our findings are depicted in Table 2. As seen, several citrullinated epitopes containing charged residues C-terminal to Cit have been described, which confirm our findings that charged amino acids have a positive influence on ACPA reactivity.

**Table 2:** Citrullinated epitopes described in the literature which contained charged amino acids in the C-terminal.

Peptide name	Origin	Sequence	Reference
Arg573	Fibrinogen α-chain	HHPGIAEFP-Cit-GKSSSYSKQF	[45]
Cit74	Fibrinogen β-chain	APPPISGGGYRA-Cit-PAKAAAT	[45]
	Fibrin α-chain	FLAEGGGV-Cit-GRPVVERH	[46]
	Fibrin β-chain	NEEGFFSA-Cit-GHRPLDKK	[46]
E12DCit	Pro-filaggrin	ESSRDGS-Cit-HPRSHD	[47]
FN-cit	Fibronectin	LTVGLT-Cit-Cit-GQPRQY	[48]
	EBNA-1	GGRRGRGRERA-Cit-GGSRERAR	[49]
	EBNA-1	ARGGSRERARGRGRG-Cit-GEKR	[49]
CCP1	Pro-filaggrin	SHQEST-Cit-GRSRGRSGRSGS	[9]

## Discussion

In the present study, we demonstrated extensive ACPA reactivity to citrullinated pro-filaggrin, originally described by Schellekens [9]. We found that factors such as peptide length, peptide conformation, peptide presentation and peptide composition notably influenced ACPA reactivity to the pro-filaggrin peptide.

The influence of peptide length and peptide conformation on ACPA reactivity was especially profound when analyzing reactivity to truncated LCP and CCP peptides. Accordingly, ACPA reactivity was obtained to **CCPa-d** compared to the non-citrullinated peptide **CCPg**, which showed a positive correlation between the number of amino acids in the cyclic structure and antibody reactivity. No notable antibody reactivity was found to peptides containing less than 8 amino acids in the cyclic structure, however, in general antibody reactivity to the cyclic peptides was reduced relative to the control for peptides containing less than 14 amino acids in the cyclic structure. These findings are in accordance to the literature, describing that antibody reactivity to citrullinated peptides smaller than 12 amino acids in length antibody reactivity is notably reduced [10,39,41]. Comparison of ACPA reactivity to the cyclic and linear peptides leave no doubt that the cyclic conformation is optimal for antibody reactivity if the peptides are smaller than 14 amino acids, whereas the full-length **CCPa** and **LCPa-B** and **CCPb** and **LCPb-B** yield similar reactivity levels. These findings contradict original findings by Schellekens *et al*, describing that **CCPa** was not more sensitive compared to **LCPa** [9].

The fact that peptide conformation was essential for ACPA reactivity, was confirmed when analyzing ACPA reactivity to D-Ala substituted peptides (Fig 4), as antibody reactivity especially the **[dA8]LCP-B** was reduced when introducing a D-amino acid, which constrains the flexibility of the peptide.

As presented in Figs 1 and 2, the biotin labelling of the linear peptides was found to be crucial for antibody reactivity. This was especially profound for the peptides with a N-terminal labelling, as only significant antibody reactivity was found to **B-LCPa**, whereas ACPA reactivity was found to peptides **LCPa-B**, **LCPb-B, LCPc-B, LCPd-B**, although only significant to peptides **LCPa-B** and **LCPb-B**. Competitive inhibition analyses, analyzing the inhibitory effect of **LCPa**, **CCPa**, **LCPb**, **CCPb**, found that the peptides were equally good at inhibiting ACPA reactivity, indicating that the reduced ACPA reactivity to **B-LCPb** and the remaining N-terminal biotinylated peptides, relates directly to the biotin labelling in the N-terminal. The fact that the positioning of the biotin label markedly influence antibody reactivity, may be explained by steric hindrance, however, this remains to be verified. Interestingly, the inhibitory effect of the biotin labelling was found to correlate with the length of the peptide, these findings are in accordance with studies by Babos and colleagues, describing anti-CCP2 reactivity to filaggrin peptides [50].

Finally, we illustrated that charged amino acids appear to be essential for ACPA reactivity, at least to the pro-filaggrin peptide examined in this study. It is tempting to speculate that this pattern relates to other citrullinated epitopes as well, as several other citrullinated epitopes have charged amino acids in the C-terminal of the epitopes as well, as presented in Table 2. The direct contribution of the charged amino acid on epitope specificity remains to be characterized in other citrullinated epitopes, currently it is only known that Cit preferably in combination with Gly or other small amino acids C-terminal to Cit are essential for ACPA reactivity [9,41].

Findings within this study are crucial in relation to peptide-based diagnostics. Several assays apply cyclic peptides, however, our results illustrate that dependent on the length and on the conformation of the peptide, different candidates are favored. Thus, although these findings confirm that peptide length and peptide conformation are essential for antibody reactivity, they do not support that all cyclic peptides necessarily are better candidates for diagnostic purposes. Another question remains whether the different peptide conformations detect varying groups of ACPA antibodies. It is tempting to speculate whether the currently applied CCP2 ELISA kit would detect higher ACPA levels if a combination of peptide conformations was applied. This remains to be determined.

In relation to the clinical diagnostics of ACPA, experiments conducted in the current study may in theory reflect the differences in diagnostic assays used for detection of ACPA. Thus, dependent on the selection of several factors such as peptide, peptide structure, assay setup, varying antibody sensitivities and specificities are obtained, which relates directly to the current assays available for detection of ACPAs. Hence, by using a gold standard for ACPA detection, it would be possible to streamline the diagnostic accuracy of ACPA detection, yielding similar ACPA sensitivities and specificities, independent of the assay applied, the place of testing and he conditions of assay testing.

Collectively, these results confirm that antibody reactivity to citrullinated pro-filaggrin seems to be defined by: peptide structure, peptide length, amino acid composition and the presence of Cit. However, these factors alone are not sufficient to detect all ACPAs, a combination of the abovementioned appear to be necessary in order to obtain maximal ACPA reactivity. Moreover, these findings illustrate how the position of biotin and most likely the distance between the biotin position and the core epitope influence ACPA reactivity.

## References

[1] Engel A, Roberts J, Burch TA. Rheumatoid arthritis in adults. Vital Health Stat 1966; 11:1–43. 5296897

[2] Sangha O. Epidemiology of rheumatic diseases. Rheumatology (Oxford) 2000; 39: S3–S12. 10.1093/rheumatology/39.suppl_2.3 11276800

[3] Pedersen JK, Kjaer NK, Svendsen AJ, Horslev-Petersen K. Incidence of rheumatoid arthritis from 1995 to 2001: impact of ascertainment from multiple sources. Rheumatol Int. 2009; 29: 411–415. 10.1007/s00296-008-0713-6 18853167

[4] Aletaha D, Neogi T, Silman AJ, Funovits J, Felson DT, Bingham CO 3rd et al Rheumatoid arthritis classification criteria: an American College of Rheumatology/European League Against Rheumatism collaborative initiative. Arthritis Rheum. 2010; 62: 2569–2581. 10.1002/art.27584 20872595

[5] Waaler E. On the occurrence of a factor in human serum activating the specific agglutintion of sheep blood corpuscles. 1939. APMIS. 2007; 115: 422–438. 10.1111/j.1600-0463.2007.apm_682a.x 17504400

[6] Visser H, Gelinck LBS, Kampfraath AH, Breedveld C, Hazes JMW. Diagnostic and prognostic characteristics of the enzyme linked immunosorbent rheumatoid arthritis assay in rheumatoid arthritis. Ann Rheum Dis. 1996; 55: 157–161. 8712877 10.1136/ard.55.3.157 PMC1010121

[7] Nell VP, Machold KP, Stamm TA, Eberl G, Heinzl H, Uffmann M et al Autoantibody profiling as early diagnostic and prognostic tool for rheumatoid arthritis. Ann Rheum Dis. 2005; 64: 1731–1736. 10.1136/ard.2005.035691 15878904 PMC1755298

[8] Shmerling RH, Delbanco TL. The rheumatoid factor: an analysis of clinical utility. Am J Med. 1991; 91: 528–534. 1951415 10.1016/0002-9343(91)90190-9

[9] Schellekens GA, Visser H, de Jong BA, van den Hoogen FH, Hazes JM, Breedveld FC et al The diagnostic properties of rheumatoid arthritis antibodies recognizing a cyclic citrullinated peptide. Arthritis Rheum. 2000; 43: 155–163. 10.1002/1529-0131(200001)43:1<155::AID-ANR20>3.0.CO;2-3 10643712

[10] Schellekens GA, de Jong BA, van den Hoogen FH, van de Putte LB, van Venrooij WJ. Citrulline is an essential constituent of antigenic determinants recognized by rheumatoid arthritis-specific autoantibodies. J Clin Invest. 1998; 101: 273–281. 10.1172/JCI1316 9421490 PMC508564

[11] Aho K, Palosuo T. Heliövaara M, Knekt P, Alha P, von Essen R. Antifilaggrin antibodies within "normal" range predict rheumatoid arthritis in a linear fashion. J Rheumatol. 2000; 27: 2743–2746. 11128658

[12] Nielen MM, van Schaardenburg D, Reesink HW, van de Stadt RJ, van der Horst-Bruinsma IE, de Koning MH et al Specific autoantibodies precede the symptoms of rheumatoid arthritis: a study of serial measurements in blood donors. Arthritis Rheum. 2004; 50: 380–386. 10.1002/art.20018 14872479

[13] Rantapaa-Dahlqvist S, de Jong BA, Berglin E, Hallmans G, Wadell G, Stenlund H et al Antibodies against cyclic citrullinated peptide and IgA rheumatoid factor predict the development of rheumatoid arthritis. Arthritis Rheum. 2003; 48: 2741–2749. 10.1002/art.11223 14558078

[14] Kastbom A, Strandberg G, Lindroos A, Skogh T. Anti-CCP antibody test predicts the disease course during 3 years in early rheumatoid arthritis (the Swedish TIRA project). Ann Rheum Dis. 2004; 63: 1085–1089. 10.1136/ard.2003.016808 15308517 PMC1755123

[15] Ronnelid J, Wick MC, Lampa J, Lindblad S, Nordmark B, Klareskog L et al Longitudinal analysis of citrullinated protein/peptide antibodies (anti-CP) during 5 year follow up in early rheumatoid arthritis: anti-CP status predicts worse disease activity and greater radiological progression. Ann Rheum Dis. 2005; 64: 1744–1749. 10.1136/ard.2004.033571 15843452 PMC1755292

[16] Chibnik LB, Mandl LA, Costenbader KH, Schur PH, Karlson EW. Comparison of threshold cutpoints and continuous measures of anti-cyclic citrullinated peptide antibodies in predicting future rheumatoid arthritis. J Rheumatol. 2009; 36: 706–711. 10.3899/jrheum.080895 19228654 PMC3108039

[17] Klareskog L, Ronnelid J, Lundberg K, Padyukov L, Alfredsson L. Immunity to citrullinated proteins in rheumatoid arthritis. Annu Rev Immunol. 2008; 26: 651–675. 10.1146/annurev.immunol.26.021607.090244 18173373

[18] Klareskog L, Stolt P, Lundberg K, Kallberg H, Bengtsson C, Grunewald J et al A new model for an etiology of rheumatoid arthritis: smoking may trigger HLA-DR (shared epitope)-restricted immune reactions to autoantigens modified by citrullination. Arthritis Rheum. 2006; 54: 38–46. 10.1002/art.21575 16385494

[19] van der Helm-van Mil AH, Verpoort KN, le Cessie S, Huizinga TW, de Vries RR, Toes RE. The HLA-DRB1 shared epitope alleles differ in the interaction with smoking and predisposition to antibodies to cyclic citrullinated peptide. Arthritis Rheum. 2007; 56: 425–432. 10.1002/art.22373 17265477

[20] Kallberg H, Padyukov L, Plenge RM, Ronnelid J, Gregersen PK, van der Helm-van Mil AH et al Epidemiological Investigation of Rheumatoid Arthritis study group. Gene-gene and gene-environment interactions involving HLA-DRB1, PTPN22, and smoking in two subsets of rheumatoid arthritis. Am J Hum Genet. 2007; 80: 867–875. 10.1086/516736 17436241 PMC1852748

[21] Wesoly J, van der Helm-van Mil AH, Toes RE Chokkalingam AP, Carlton VE, Begovich AB et al Association of the PTPN22 C1858T single-nucleotide polymorphism with rheumatoid arthritis phenotypes in an inception cohort. Arthritis Rheum. 2005; 52: 2948–2950. 10.1002/art.21294 16145680

[22] Pedersen M, Jacobsen S, Garred P, Madsen HO, Klarlund M, Svejgaard A et al Strong combined gene-environment effects in anti-cyclic citrullinated peptide-positive rheumatoid arthritis: a nationwide case-control study in Denmark. Arthritis Rheum. 2007; 56: 1446–1453. 10.1002/art.22597 17469102

[23] Bartold PM, Marino V, Cantley M, Haynes DR. Effect of Porphyromonas gingivalis-induced inflammation on the development of rheumatoid arthritis. J Clin Periodontol. 2010; 37: 405–411. 10.1111/j.1600-051X.2010.01552.x 20507365

[24] Burkhardt H, Sehnert B, Bockermann R, Engstrom A, Kalden JR, Holmdahl R. Humoral immune response to citrullinated collagen type II determinants in early rheumatoid arthritis. Eur J Immunol. 2005; 35: 1643–1652. 10.1002/eji.200526000 15832289

[25] Kinloch A, Tatzer V, Wait R, Peston D, Lundberg K, Donatien P et al Identification of citrullinated alpha-enolase as a candidate autoantigen in rheumatoid arthritis. Arthritis Res Ther. 2005; 7: R1421–R1429. 10.1186/ar1845 16277695 PMC1297593

[26] Snir O, Widhe M, von Spee C, Lindberg J, Padyukov L, Lundberg K et al Multiple antibody reactivities to citrullinated antigens in sera from patients with rheumatoid arthritis: association with HLA-DRB1 alleles. Ann Rheum Dis. 2009; 68: 736–743. 10.1136/ard.2008.091355 18635594

[27] Vossenaar ER, Despres N, Lapointe E, van der Heijden A, Lora M, Senshu T et al Rheumatoid arthritis specific anti-Sa antibodies target citrullinated vimentin. Arthritis Res Ther. 2004; 6: R142–R150. 10.1186/ar1149 15059278 PMC400433

[28] Takizawa Y, Suzuki A, Sawada T, Ohsaka M, Inoue T, Yamada R et al Citrullinated fibrinogen detected as a soluble citrullinated autoantigen in rheumatoid arthritis synovial fluids. Ann Rheum Dis. 2006; 65: 1013–1020. 10.1136/ard.2005.044743 16449316 PMC1798256

[29] Tarcsa E, Marekov LN, Mei G, Melino G, Lee SC, Steinert PM. Protein unfolding by peptidylarginine deiminase. Substrate specificity and structural relationships of the natural substrates trichohyalin and filaggrin. J Biol Chem. 1996; 271: 30709–30716. 8940048 10.1074/jbc.271.48.30709

[30] Girbal-Neuauser E, Durieux JJ, Arnaud M, Dalbon P, Sebbag M, Vincent C et al The epitopes targeted by the rheumatoid arthritis-associated antifilaggrin autoantibodies are posttranslationally generated on various sites of (pro)filaggrin by deimination of arginine residues. J Immunol 1999; 162: 585–594. 9886436

[31] Simon M, Girbal E, Sebbag M, Gomès-Daudrix V, Vincent C, Salama G et al The cytokeratin filament-aggregating protein filaggrin is the target of the so-called "antikeratin antibodies," autoantibodies specific for rheumatoid arthritis. J Clin Invest. 1993; 92: 1387–1393. 10.1172/JCI116713 7690781 PMC288281

[32] Sebbag M, Simon M, Vincent C, Masson-Bessière C, Girbal E, Durieux JJ et al The antiperinuclear factor and the so-called antikeratin antibodies are the same rheumatoid arthritis-specific autoantibodies. J Clin Invest. 1995; 95: 2672–2679. 10.1172/JCI117969 7539459 PMC295950

[33] van Venrooij WJ, van Beers JJ, Pruijn GJ. Anti-CCP Antibody, a Marker for the Early Detection of Rheumatoid Arthritis. Ann NY Acad Sci. 2008; 1143:268–285. 10.1196/annals.1443.013 19076355

[34] Li SC, Kim PK, Deber CM. Manipulation of peptide conformations by fine-tuning of the environment and/or the primary sequence. Biopolymers. 1995; 35: 667–675. 10.1002/bip.360350612 7766831

[35] Williamson MP, Handa BK, Hall MJ. Secondary structure of a herpes simplex virus glycoprotein D antigenic domain. Int J Pept Protein Res. 1986; 27: 562–568. 2426211

[36] Dyson HJ, Wright PE. Antigenic peptides. FASEB J. 1995; 9:37–42. 7821757 10.1096/fasebj.9.1.7821757

[37] Nair DT, Singh K, Siddiqui Z, Nayak BP, Rao KV. Salunke D.M. Epitope recognition by diverse antibodies suggests conformational convergence in an antibody response. J Immunol. 2002; 168, 2371–2382. 11859128 10.4049/jimmunol.168.5.2371

[38] Dorow DS, Shi PT, Carbone FR, Minasian E, Todd PE, Leach SJ. Two large immunogenic and antigenic myoglobin peptides and the effects of cyclisation. Mol Immunol. 1985; 22: 255–264. 4079944 10.1016/0161-5890(85)90044-6

[39] Kobylyansky AG, Nekrasov AN, Kozlova VI, Sandin MY, Alikhanov BA, Demkin VV. Detection of new epitopes of antibodies to filaggrin in filaggrin protein molecule. Bull Exp Biol Med. 2011; 151: 615–618. 22462059 10.1007/s10517-011-1396-7

[40] Soutullo A, Santi MN, Perin JC, Beltramini LM, Borel IM, Frank R et al Systematic epitope analysis of the p26 EIAV core protein. J Mol Recognit. 2007; 20: 227–237. 10.1002/jmr.825 17705340

[41] Trier NH, Leth M, Hansen PR, Houen G. Cross-reactivity of a human IgG_1_ anti-citrullinated fibronogen monoclonal antibody to a citrullinated pro-filaggrin peptid. Pro Sci. 2012; 21: 1929–1941. 10.1002/pro.2178 PMC3575922 23076998

[42] Trier NH, Hansen PR, Vedeler CA, Somnier FE, Houen G. Identification of continuous epitopes of HuD antibodies related to paraneoplastic diseases/small cell lung cancer. J Neuroimmunol. 2012; 243: 25–33. 10.1016/j.jneuroim.2011.12.020 22264992

[43] Arnett FC, Edworthy SM, Bloch DA, McShane DJ, Fries JF, Cooper NS et al The American Rheumatism Association 1987 revised criteria for the classification of rheumatoid arthritis. Arthritis Rheum. 1988; 31: 315–24. 3358796 10.1002/art.1780310302

[44] Mahalakshmi R, Balaram P. The use of D-amino acids in peptide design D-Amino Acids: A New Frontier in Amino Acid and Protein Research—Practical Methods and Protocols. 2006; 415–430. Nova science publishers.

[45] Fernandes-Cerqueira C, Ossipova E, Gunasekera S, Hansson M, Mathsson L, Catrina AI et al Targeting of anti-citrullinated protein/ peptide antibodies in rheumatoid arthritis using peptides mimicking endogenously citrullinated fibrinogen antigens. Arthritis Res Ther. 2015;17:155 10.1186/s13075-015-0666-6 26059223 PMC4484629

[46] Van der Woude D, Rantapää-Dahlqvist S, Ioan-Facsinay A, Onnekink C, Schwarte CM, Verpoort KN et al Epitope spreading of the anti-citrullinated protein antibody response occurs before disease onset and is associated with the disease course of early arthritis. Ann Rheum Dis. 2010; 69, 8: 1554–61. 10.1136/ard.2009.124537 20448290

[47] Girbal-Neuhauser E, Durieux J J, Arnaud M, Dalbon P, Sebbag M, Vincent C et al The epitopes targeted by the rheumatoid arthritis-associated antifilaggrin autoantibodies are posttranslationally generated on various sites of (pro)filaggrin by deimination of arginine residues. J Immunol. 1999; 162: 585–594. 9886436

[48] Beers JJ, Willemze A, Stammen-Vogelzangs J, Drijfhout JW, Toes RE, Pruijn GJ. Anti-citrullinated fibronectin antibodies in rheumatoid arthritis are associated with human leukocyte antigen-DRB1 shared epitope alleles. Arthritis Res Ther. 2012; 14: R35 10.1186/ar3744 22339947 PMC3392834

[49] Trier NH, Holm BE, Slot O, Locht H, Lindegaard H, Svendsen A et al Application of synthetic peptides for detection of anti-citrullinated peptide antibodies. Peptides 2016; 76: 87–95. 10.1016/j.peptides.2016.01.005 26796582

[50] Babos F, Szarka E, Nagy G, Majer Z, Sármay G, Magyar A et al Role of N or C-terminal biotinylation in autoantibody recognition of citrullin containing filaggrin epitope peptides in rheumatoid arthritis. Bioconjug Chem. 2013; 24: 817–827. 10.1021/bc400073z 23617702

